# Integrative analysis of sensory evaluation and non-targeted metabolomics to unravel tobacco leaf metabolites associated with sensory quality of heated tobacco

**DOI:** 10.3389/fpls.2023.1123100

**Published:** 2023-02-08

**Authors:** Lu Zhao, Shanzhai Shang, Yongfeng Tian, Yulong Gao, Zhongbang Song, Lijuan Peng, Zhuolin Li, Bingwu Wang

**Affiliations:** ^1^ National Tobacco Genetic Engineering Research Center, Yunnan Academy of Tobacco Agricultural Sciences, Kunming, Yunnan, China; ^2^ Research and Development Center, China Tobacco Yunnan Industrial Co., Ltd., Kunming, Yunnan, China; ^3^ Laboratory of Tobacco Chemistry, Yunnan Tobacco Quality Supervision and Test Station, Kunming, Yunnan, China; ^4^ Department of Technical Support, Malong Branch of Qujing Tobacco Company, Qujing, Yunnan, China

**Keywords:** heated tobacco products, sensory evaluation, non-targeted metabolomics profiling, orthogonal projections to latent structures discriminant analysis, discriminating metabolites

## Abstract

**Introduction:**

Heated tobacco (*Nicotiana tabacum* L.) products are heating tobacco plug at a temperature of 350°C and produce different emissions in aerosol and sensory perceptions of tobacco leaf compared with combustible tobacco. Previous study assessed different tobacco varieties in heated tobacco for sensory quality and analyzed the links between sensory scores of the final products and certain chemical classes in tobacco leaf. However, contribution of individual metabolites to sensory quality of heated tobacco remains largely open for investigation.

**Methods:**

In present study, five tobacco varieties were evaluated as heated tobacco for sensory quality by an expert panel and the volatile and non-volatile metabolites were analyzed by non-targeted metabolomics profiling.

**Results:**

The five tobacco varieties had distinct sensory qualities and can be classified into higher and lower sensory rating classes. Principle component analysis and hierarchical cluster analysis showed that leaf volatile and non-volatile metabolome annotated were grouped and clustered by sensory ratings of heated tobacco. Orthogonal projections to latent structures discriminant analysis followed by variable importance in projection and fold-change analysis revealed 13 volatiles and 345 non-volatiles able to discriminate the tobacco varieties with higher and lower sensory ratings. Some compounds such as β-damascenone, scopoletin, chlorogenic acids, neochlorogenic acids, and flavonol glycosyl derivatives had strong contribution to the prediction of sensory quality of heated tobacco. Several *lyso*-phosphatidylcholine and *lyso*-phosphatidylethanolamine lipid species, and reducing and non-reducing sugar molecules were also positively related to sensory quality.

**Discussion:**

Taken together, these discriminating volatile and non-volatile metabolites support the role of leaf metabolites in affecting the sensory quality of heated tobacco and provide new information on the types of leaf metabolites that can be used to predict applicability of tobacco varieties for heated tobacco products.

## 1 Introduction

Sensory quality of tobacco products is perceived through organoleptic attributes including aroma and mouthful feeling, and is generally assessed by experts who use all senses and professional judgements to identify the aroma types, recognize the flaws in taste, and determine the suitability in making tobacco blends (Carpenter et al., 2007). Sensory attributes such as aroma and mouthfeel sensation of combustible tobacco are predominantly determined by the chemical compositions of tobacco leaf (Weeks et al., 1992). Oronasal aroma is influenced by volatile metabolites and volatile precursors, which mainly impart the typical tobacco flavor and pleasant aroma (Sun et al., 2011; Popova et al., 2019). While the mouthfeel sensation is mainly affected by non-volatile metabolites. For example, organic acids and nicotine were found to make positive contributions to the taste and strength characteristics of tobacco product, respectively (Wang et al., 2008). Non-volatile carbonyls and unsaturated hydrocarbons were reported to enhance taste and contribute to quality (Mendell et al., 2014; Banožić et al., 2020). In addition, chemical compositions, and interaction between volatile and non-volatile compounds also affect the sensory properties of tobacco products. For example, flue-cured tobacco cultivar ‘Zhongyantexiang 301’ was perceived to produce heady rose-like aroma in the sensory evaluation of combustible tobacco, and its aroma characteristic was found to strongly associate with higher concentrations of reducing sugars, β-damascenone, trans-β-ocimene, and a higher sugar to alkaloid ratio in the cured leaf (Xu et al., 2019).

Heated tobacco (*Nicotiana tabacum* L.) is a new type of tobacco product that relies on a battery powered heating system to heat a tobacco plug in the cartridge to around 200-350°C and trigger aerosol production containing nicotine and aroma substances (Simonavicius et al., 2019). The tobacco plug is made from tobacco leaves that are grounded and reconstituted into sheets with the addition of flavors or additives, wood cellulose fibers and aerosol formers (e.g. glycerin, propylene glycol and ethylene glycol) (Czegeny et al., 2016; Smith et al., 2016). As tobacco combustion is eliminated (Eaton et al., 2018), tobacco pyrolysis in heated tobacco product is reduced to a minimum and aerosol formation mainly undergo evaporation and distillation (Schaller et al., 2016; Gasparyan et al., 2018), resulting in significant reduction of harmful constituents such as tar and carbon monoxide in the mainstream smoke (Farsalinos et al., 2018). In contrast to a greater than 800°C combustion in burning tobacco, heating at a temperature around 350°C and different leaf processing presumably lead to different emission profiles and sensory perceptions of tobacco varieties in heated tobacco products (Cancelada et al., 2019). One study of five flue-cured tobacco varieties showed that Yunyan 116, an industrial-favored flue-cured tobacco variety in manufacturing combustible tobacco products, was not highly rated when used in heated tobacco products (Zhao et al., 2020).

Tobacco leaf chemistry and their correlations with the aroma and sensory quality of combustible tobacco have been investigated (Weeks et al., 1992; Carpenter et al., 2007). Many aroma-related components were used as chemical indicators for better sensory perception or identified as breeding targets relating to quality traits (Cui et al., 2011; Zhang et al., 2015; Zhang et al., 2018). With respect to the heated tobacco products, aroma-related markers that were identified for combustible tobacco were utilized in the prediction of sensory quality at low heating temperatures, and the sum of concentrations of all individual metabolites of certain chemical classes (e.g. polyphenols, aldehydes, ketones, and alcohols) were used to characterize their associations with the sensory scores of final products (Chen et al., 2021). However, compounds of the same chemical class are likely to contribute to and/or detract from sensory quality, and many could have little or no impact (Chen et al., 2021), and combustible and heated tobacco may have different suites of significant metabolites associated with sensory quality due to different working temperatures. Consequently, difficulties in interpretating the relationships between these compounds and sensory properties of heated tobacco were encountered (Zhao et al., 2020; Chen et al., 2021).

Metabolomics profiling has been widely applied in food industry and multivariate analysis such as partial least squares regression (PLSR) and orthogonal projections to latent structures discriminant analysis (OPLS-DA) were commonly used to identify significant flavor metabolites that vary in samples with different sensory traits (Schmidtke et al., 2013; Romanini et al., 2019; Sherman et al., 2020). To better understand the relationships between tobacco leaf metabolites and sensory quality of heated tobacco, and identify significant metabolites that can be used in prediction of sensory quality, volatile and non-volatile metabolomes of five tobacco varieties were acquired using non-targeted metabolomics profiling and used to predict sensory quality ratings as assessed by a panel of experts in this study. Discriminating compounds contributing to the prediction of sensory quality of heated tobacco were identified by OPLS-DA. Five tobacco varieties planted and cured in Yunnan province in China were selected on the basis of employing different curing practices after field harvest, normally being used in combustible tobacco products such as traditional blended cigarettes and pipe tobacco. This study tested the hypothesis that the tobacco leaf metabolites influenced the sensory quality of heated tobacco in a way that allow the metabolomics profiling and OPLS-DA to identify significant metabolites with utility in prediction of sensory quality.

## 2 Materials and methods

### 2.1 Plant materials

Five tobacco varieties produced in Yunnan province, including flue-cured tobacco K326, sun-cured tobaccos Luxitu (LXT) and Luxilao (LXL), sun/air-cured tobaccos Leye (LY) and Badahe (BDH), were evaluated for the applicability to heated tobacco products. The middle tobacco leaves were harvested 3 weeks post topping and processed as previously described in Chen et al. (2021).

### 2.2 Preparation of heated tobacco cartridge and sensory evaluation

The cured leaves of each tobacco variety were processed to manufacture the heated tobacco cartridges as described in Chen et al. (2021). Briefly, the cured leaf was ground into powder and added into the coating slurry used to manufacture the reconstituted tobacco sheet. The coating slurry contained 10% of tobacco leaf powder and 90% of the mixture of glycerin, propylene glycol and exogenous wood pulp fibers. The reconstituted sheet was then shredded, rolled in bundles, and made into the tobacco plug. The tobacco cartridge was heated in the *Webacco* holder (China Tobacco Yunnan Industrial Co., Ltd.) at the temperature of 350°C.

Eleven qualified experts who had more than five years’ experience in tobacco industry (China) were recruited to undertake the descriptive sensory and quality evaluations of heated tobacco samples based on the Sensory Evaluation Standard of Heated Tobacco Products as described in Chen et al. (2021). Panelists were asked to rate each heated tobacco sample on the intensities of six sensory attributes including volume of smoke (10 scores), aroma (30 scores), physiological strength (10 scores), coordination (10 scores), irritation (15 scores), and taste (25 scores). The overall sensory quality (100 scores) was rated last based on their own concepts. The score of each attribute was calculated using the formula ${\bar{X}}_{i},=\Sigma X_{i}/n,$ where ${\bar{X}}_{i},\Sigma X_{i}$ and n represent the mean score, the sum of scores and the number of participants, respectively (Chen et al., 2021). Tobacco samples were heated using the same carrier and evaluated by the same panel of experts, and then ranked by the overall sensory scores to categorize the tobacco varieties into “higher” or “lower” sensory rating class. The point 85 was used to sort the tobacco varieties, in which an overall sensory score higher than 85 for higher sensory rating class, and a score lower than 85 for lower sensory rating class. The rationale was that metabolome analysis was performed to profile the cured leaves of five tobacco varieties and only the leaf metabolites that differ between higher and lower sensory rating classes were focused on characterization.

### 2.3 Volatile profiling of tobacco leaf

Cured leaf samples were taken at the time of preparing heated tobacco cartridge and stored at -80°C until analysis. Leaf samples were ground into fine powder using liquid nitrogen and an aliquot (1 gram) of leaf powder was transferred immediately to a 20 mL head-space vial (Agilent, Palo Alto, CA, United States), containing NaCl saturated solution to inhibit any enzyme reaction. The vials were sealed with crimp-top caps with TFE-silicone headspace septa (Agilent). The solid phase microextraction (SPME) analysis and identification of volatile metabolites were performed as previously described (Yuan et al., 2022).Mass spectra were analyzed using Agilent MassHunter software (Agilent Technologies, Santa Clara, CA, USA). Volatile compounds were identified by comparison of mass spectra to those in the NIST library and an in-house Metware database (WMDB) (Wei et al., 2016; Peng et al., 2022).

### 2.4 Non-volatile profiling of tobacco leaf

Cured leaf samples were taken at the time of preparing heated tobacco cartridge, and then lyophilized using a vacuum freeze-dryer (Scientz-100F) and ground using a mixer mill (MM400, Retsch) with a zirconia bead for 1.5 min at 30Hz. An aliquot (100 mg) of leaf powder was suspended in 1.2 mL of 70% methanol solution, vortexed for 30 s at 30 min intervals for six times in total, then incubated at 4°C for 12 h. Following incubation, samples were centrifuged at 12,000 rpm for 10 min, and the extracts were filtrated (SCAA-104, 0.22 μm pore size; ANPEL, Shanghai, China) prior to ultra performance liquid chromatography-tandem mass spectrometry (UPLC-MS/MS) analysis. Non-volatile metabolite profiling and data processing were performed as previously described (Chen et al., 2013). Quantification and annotation of non-volatile metabolites were achieved through a scheduled multiple reaction monitoring method (Zou et al., 2020).

### 2.5 Data analysis

The ratings of sensory attributes and overall quality were analyzed by a univariate analysis of variance (ANOVA) where tobacco variety was considered as a fixed effect while panelist was considered as a random effect. Tukey’s honest significant difference (HSD) test was used as a *post-hoc* comparison of means.

Unsupervised principal component analysis (PCA) is designed to reduce the dimensionality of the original data, extract sample composition information and remove noise (Liland, 2011; Mascellani et al., 2021). To analyze the difference in chemical composition among different tobacco varieties, PCA was conducted on unit-variance (UV) scaled metabolites with SIMCA software 14.1 (Umetrics, Sartorius Stedim Biotech, Umea, Sweden) (Wang et al., 2020a). Hierarchical cluster analysis (HCA) is an unsupervised method that groups data according to their affinity in clusters of progressive dissimilarity (Mascellani et al., 2021) and presented as the heatmap with dendrogram. Volatile and non-volatile metabolite data were normalized *via* z-transformation and then converted into colors and grouped using hierarchical clustering (Cheadle et al., 2003). The hierarchically clustered heatmaps were generated using MetaboAnalyst 5.0 with default settings (Pang et al., 2022). The similarity among group averages was measured using the Pearson distance and the cluster aggregation was based on the average linkage method.

Orthogonal projections to latent structures discriminant analysis (OPLS-DA), performed in SIMCA software 14.1 (Umetrics, Sartorius Stedim Biotech, Umea, Sweden), was conducted to separate systematic variation based on linearity and orthogonality, and extract significant metabolites that discriminate sample groups (Wang et al., 2020a). The quality of model was examined by checking the values of R^2^Y (goodness-of fit) and Q^2^Y (goodness of prediction), and a value > 0.5 was adopted as the threshold of acceptable models (Romanini et al., 2019). Permutation testing was performed for 200 times to avoid overfitting, and the model is reliable when the R^2^ > 0 and Q^2^< 0. Variable importance in projection (VIP) was used to identify volatile and non-volatile metabolites with highest discrimination potential (VIP score > 1.0). The discriminant metabolites with VIP > 1 and p-value< 0.05 were subjected to fold-change (cut off > 2.0 or< 0.5) analysis.

## 3 Results

### 3.1 Sensory evaluation of five tobacco varieties

The sensory quality was assessed by 11 panelists for the scores of six sensory attributes and overall quality (**Table 1**). The scores of aroma, coordination, irritation, taste and sensory quality were significantly different among tobacco varieties. The scores of overall sensory quality were not significantly different among K326, LXT and LXL, but they scored higher than LY and BDH. There were no significant effects on the attributes of smoke volume and physiological strength due to tobacco varieties but there were differences in scores of physiological strength based on tobacco varieties. The overall sensory quality varied among tobacco varieties. Sun-/air-cured tobacco varieties LY and BDH were rated lower in sensory quality than sun-cured tobacco varieties LXL and LXT, and flue-cured tobacco K326.

**Table 1:** Comparison of the mean scores of six sensory attributes and overall quality for five tobacco varieties in sensory evaluation.

Tobacco variety	Smoke volume (10)	Aroma (30)	Physiological strength (10)	Coordination (10)	Irritation (15)	Taste (25)	Sensory quality (100)
K326	8.50	25.17 ^ab^	8.17	9.00 ^a^	12.17 ^ab^	22.33 ^ab^	86.33 ^a^
LXT	8.50	25.00 ^bc^	8.17	8.33 ^ab^	12.33 ^a^	22.67 ^a^	85.50 ^a^
LXL	8.50	26.00 ^a^	8.33	8.33 ^ab^	12.17 ^ab^	22.17 ^ab^	86.33 ^a^
LY	8.50	24.67 ^bc^	7.67	7.67 ^b^	11.67 ^ab^	21.67 ^b^	82.17 ^b^
BDH	8.50	24.17 ^c^	8.63	7.63 ^b^	11.33 ^b^	21.83 ^ab^	82.00 ^b^

Table values are the measured means.

Unique lowercase letters within the same column indicate significant differences between different tobacco varieties, p< 0.05.

### 3.2 Volatile and non-volatile metabolomics profiling of five tobacco varieties

**Figure 1:**
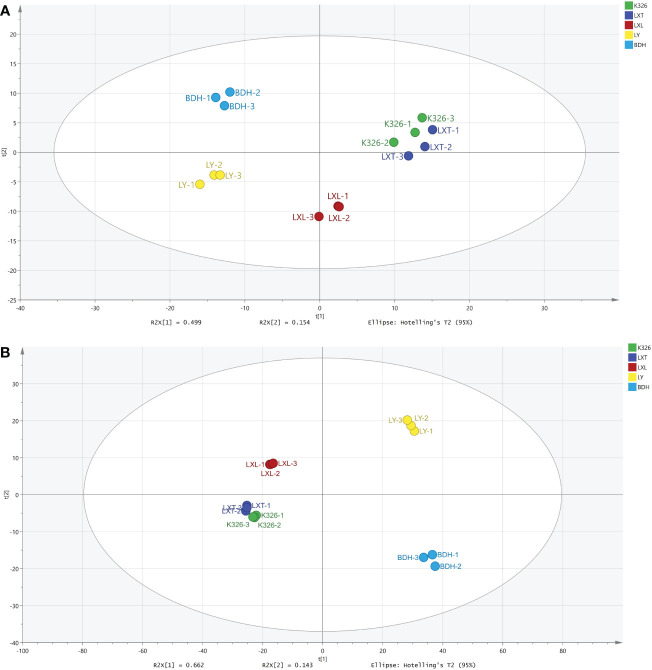
Metabolite variation among five tobacco varieties. **(A)** PCA score plot for the 309 annotated volatile metabolites (R^2^X = 0.923, Q^2^ = 0.783). **(B)** PCA score plot for the 1168 annotated non-volatile metabolites (R^2^X = 0.932, Q^2^ = 0.871).

To investigate whether the differences in sensory qualities were attributed to metabolite chemistry, metabolomics profiling was performed for volatiles and non-volatiles using GC-MS and UPLC-MS/MS, respectively. Full dataset multivariate unsupervised statistical analysis through PCA was performed on volatile and non-volatile profiling data, respectively (**Supplementary Tables S1**, **S2**). A five-component PCA model was obtained for 309 volatile metabolites with the goodness of fit (R^2^X) and predictive power (Q2) both greater than 0.75 (**Figure 1A**). The PCA score plot of 309 volatiles showed three separated clusters, with the first cluster describing K326 and LXT, the second cluster describing LXL, and the third cluster containing LY and BDH. PC1 (49.9% of the variation) was attributed to the distinct volatile profiles of LY and BDH, and PC2 (15.4% of the variation) was associated with differences in LXL. For the 1168 non-volatile metabolites, a four-component PCA model was obtained with R^2^X = 0.932 and Q^2^ = 0.871, and PC1 (66.2% of the variation) demonstrated separation between higher (K326, LXT and LXL) and lower (LY and BDH) sensory rating varieties (**Figure 1B**).

**Figure 2:**
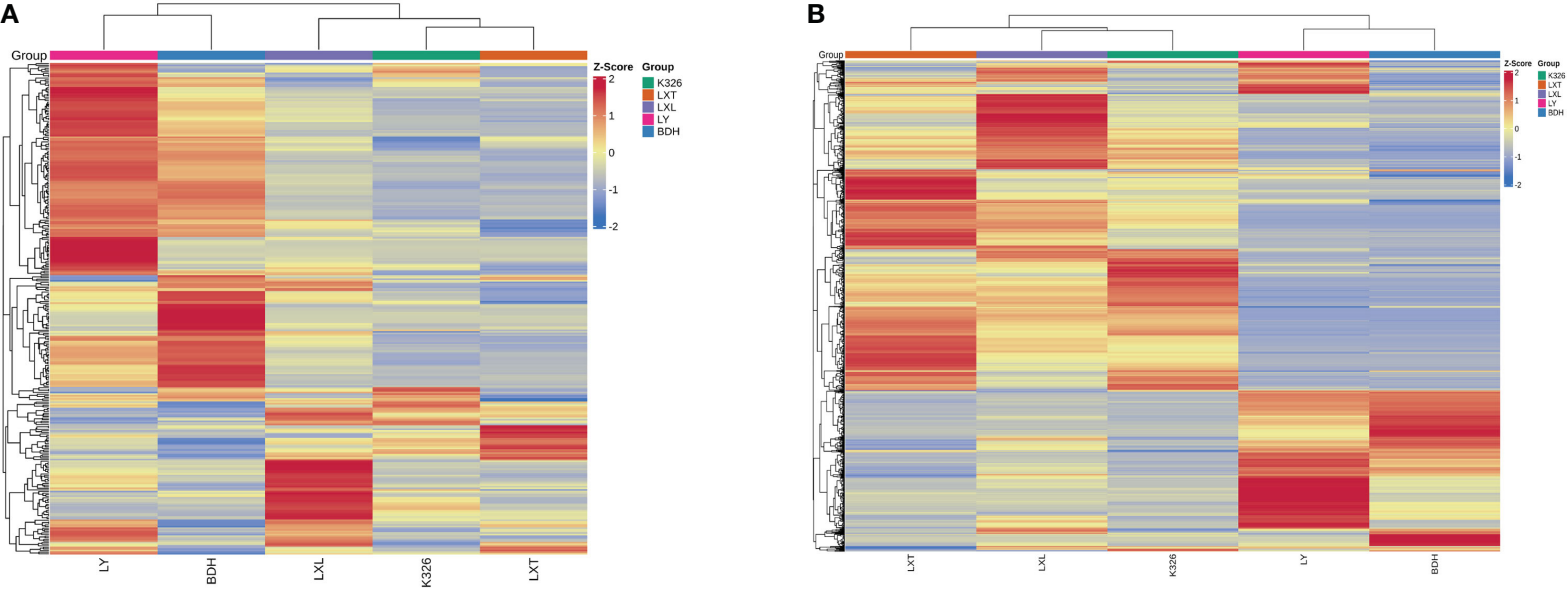
Hierarchical clustering of volatile **(A)** and non-volatile **(B)** profiles of tobacco leaf by variety. Volatile and non-volatile metabolite data were normalized within each variety via z-transformation. The resulting z-scores were converted into colors and grouped using hierarchical clustering. The color in each cell represents the z-transformed abundances of the averaged replicates (n = 3) per tobacco variety (red: high, blue: low).

Hierarchical clustering analysis and heatmap revealed that five tobacco varieties can be classified into two distinct clades based on the signal intensities of the volatile metabolites and/or non-volatile metabolites. One clade contained K326, LXL and LXT, and the other clade included LY and BDH (**Figure 2**). In the heatmap of volatiles, K326 and LXT belonged to the same sub-clade and were clustered with LXL (**Figure 2A**). By contrast, the heatmap of non-volatiles showed that K326 belonged to the same sub-clade as LXL and then clustered with LXT (**Figure 2B**). The PCA and clustering results demonstrated that the higher sensory rated varieties K326, LXL and LXT had similar metabolite profiles of volatiles and non-volatiles, and their metabolomic profiles differed from the lower sensory rated varieties LY and BDH. Therefore, it is likely that specific chemical compositions might associate with perceived sensory quality.

### 3.3 OPLS-DA modeling characterized leaf volatile and non-volatile metabolites that co-varied with overall sensory quality of heated tobacco

**Figure 3:**
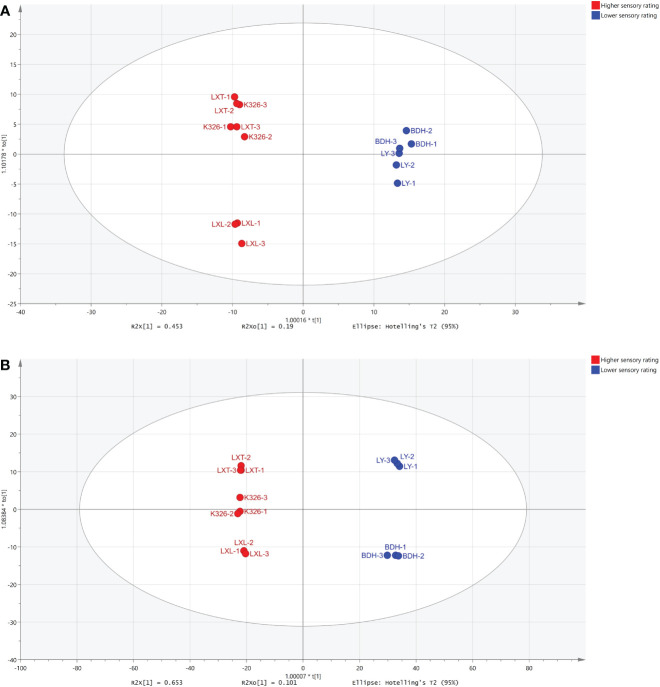
OPLS-DA performed on **(A)** 309 volatile metabolites and **(B)** 1168 non-volatile metabolites identified in five tobacco varieties according to the higher and lower sensory ratings. The colors indicate the different levels of sensory ratings (red: higher sensory rating, blue: lower sensory rating).

To investigate relationship between leaf metabolite profiles and overall sensory quality, an OPLS-DA supervised approach was performed on 309 volatile and/or 1168 non-volatile metabolites according to the sensory ratings of the tobacco varieties. The OPLS-DA models developed with volatile metabolites and/or non-volatile metabolites provided strong clustering according to the sensory ratings (**Figure 3**). The R^2^Y and Q^2^Y of both models were 0.99 and 0.99, respectively. The R^2^X being correlated to Y were 0.45 and 0.65, respectively, for the volatiles (**Figure 3A**) and non-volatiles (**Figure 3B**), hence much higher than the orthogonal (uncorrelated to Y) components which were 0.19 and 0.10, respectively. These results demonstrated that the sensory ratings mainly accounted for the overall variability observed in the metabolite dataset (volatile and non-volatile compounds) and chemical indicators of overall sensory quality, unrelated to a specific tobacco variety, could be identified.

**Figure 4:**
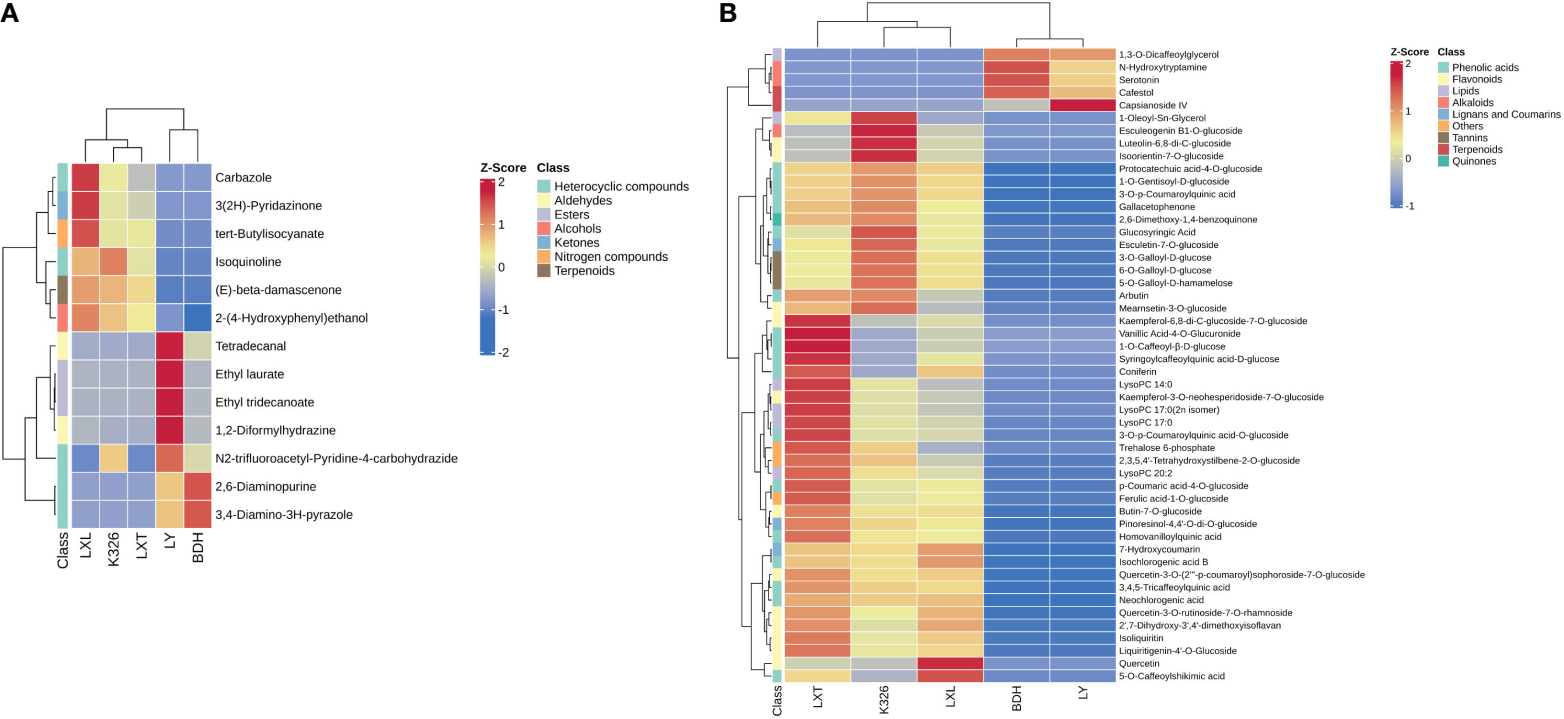
Analysis of metabolite variation among five tobacco varieties within chemical classes. **(A)** Heatmap of 13 volatile metabolites including one alcohol, two aldehydes, two esters, five heterocyclic compounds, one ketone, one nitrogen compound and one terpenoid. **(B)** Heatmap of the top 50 discriminating non-volatile compounds ranked by t-test including three alkaloids, 12 flavonoids, three lignans and coumarins, six lipids, 17 phenolic acids, three tannins, two terpenoids, one quinone, one saccharide and two other non-volatile metabolites. Volatile and non-volatile metabolite data were normalized within each variety via z-transformation. The resulting z-scores were converted into colors and grouped using hierarchical clustering. The color in each cell represents the z-transformed abundances of the averaged replicates (n = 3) per tobacco variety (red: high, blue: low).

Volatile and non-volatile compounds possessing the highest discrimination capacity (VIP > 1.0 and p-value< 0.01) between higher and lower sensory ratings were identified, regardless of the type of tobacco variety (**Supplementary Tables S3**, **S4**). Discriminating volatile and non-volatile compounds correlated with the higher or lower sensory ratings were further identified with fold-change values (fold-change cut off > 1.5 or< 0.5 for volatiles; fold-change cut off > 2 or< 0.5 for non-volatiles). Volatile compounds such as alcohols, aldehydes, esters, heterocyclic compounds, ketones, nitrogen compounds and terpenoids contributed to the prediction of sensory quality of heated tobacco products (**Table 2**). Metabolite abundances were z-transformed and the colors denote range in variation of a compound class within a variety, with red (high) and blue (low) indicate proportions of a compound’s contribution to the profile (**Figure 4**). Grouping of tobacco varieties based on 13 volatile compounds revealed two distinct clades, with one clade containing LXL, K326 and LXT, and the other clade containing LY and BDH (**Figure 4A**). Relatively higher abundances of carbazole, 3(2H)-pyridazinone, tert-butylisocyanate, isoquinoline, (E)-beta-damascenone, 2-(4-hydroxyphenyl) ethanol were observed in the higher sensory rating varieties LXL, K326 and LXT.

**Table 2:** Discriminating volatile compounds identified by VIP analysis and p-value together with fold-change cut off > 1.5 or < 0.5.

Compound ID	Class	Compounds	CAS	VIP scores	p-value (higher *vs*. lower)	Log_2_FC normalized (higher *vs*. lower)
NMW0193	Alcohol	2-(4-Hydroxyphenyl)ethanol	501-94-0	1.06	0.004	-1.17
KMW0640	Aldehyde	Tetradecanal	124-25-4	1.40	3.25×10^–8^	0.61
XMW0908	Aldehyde	1,2-Diformylhydrazine	628-36-4	1.10	0.001	1.84
KMW0594	Ester	Ethyl laurate	106-33-2	1.13	0.001	1.97
XMW1359	Ester	Ethyl tridecanoate	28267-29-0	1.44	2.40×10^–10^	2.21
NMW0107	Heterocyclic compound	Isoquinoline	119-65-3	1.48	7.31×10^–19^	-2.29
NMW0367	Heterocyclic compound	Carbazole	86-74-8	1.47	1.63×10^–14^	-2.26
XMW2892	Heterocyclic compound	N2-Trifluoroacetyl-pyridine-4-carbohydrazide	332055-87-5	1.10	0.001	2.11
XMW0335	Heterocyclic compound	3,4-Diamino-3H-pyrazole	1000288-40-0	1.48	3.64×10^–22^	2.39
XMW2604	Heterocyclic compound	2,6-Diaminopurine	1904-98-9	1.48	2.59×10^–20^	2.19
XMW0886	Ketone	3(2H)-Pyridazinone	504-30-3	1.48	1.20×10^–15^	-2.29
XMW2507	Nitrogen compounds	tert-Butylisocyanate	1609-86-5	1.48	1.97×10^–16^	-2.00
KMW0526	Terpenoids	(E)-beta-damascenone	23726-93-4	1.48	8.69×10^–24^	-2.62

Non-volatile compounds including 21 alkaloids, four amino acids and derivatives, 21 lignans and coumarins, 43 lipids, 15 nucleotides and derivatives, 117 flavonoids, six organic acids, 82 phenolic acids, two quinones, five tannins, five terpenoids and 24 others were characterized with higher VIP scores (VIP > 1.0) and strongly correlated with higher and lower sensory ratings (p-value< 0.01, fold-change > 2) (**Supplementary Table S5**). The heatmap displaying the top 50 discriminating non-volatile compounds ranked by t-test exhibited two distinct clades corresponding to tobacco varieties with relatively higher sensory ratings (LXT, K326 and LXL) and those with lower sensory ratings (LY and BDH) (**Figure 4B**). In the resulting heatmap of non-volatile metabolites including alkaloids, amino acids, nucleotides and derivatives (**Supplementary Figure S1**), BDH and LY had similar profiles and were distinct from LXL, K326 and LXT. BDH and LY had higher abundances of serotonin, N-hydroxytryptamine, hexanoyl-L-glycine, L-sepiapterin, 2’-deoxycytidine-5’-monophosphate and 1-methylxanthine, while LXL, K326 and LXT had overall reduced abundances of these compounds. The most distinct nucleotides and derivatives in LY were 8-hydroxy-2-deoxyguanosine and 2’-deoxyadenosine. For the rest of the identified alkaloids, amino acids, nucleotides and derivatives, BDH and LY had overall reduced abundances, whereas LXL, K326 and LXT had more distinct profiles. A similar pattern was observed for lignans, quinones, terpenoids and other compounds, where BDH and LY had lower abundances of most compounds in these classes, with the exceptions of piperitol, fraxetin-7,8-di-O-glucoside, cafestol and 3’-acetylsweroside (**Supplementary Figure S2**). LXL, K326 and LXT lignan profiles were higher in several coumarins such as scopoletin-7-O-glucoside, scopoletin β-D-glucuronide, scopoletin-7-O-xylosyl (1→6) glucoside and isoscopoletin. The abundances of several di-, tri- and oligosaccharides (i.e., D-sucrose, galactinol, trehalose 6-phosphate, D-melezitose, lactobiose, D-lactulose, raffinose, D-melezitose O-rhamnoside, etc.) were higher in LXT and K326, while lower in LXL, BDH and LY.

In the heatmap of 43 lipids, several lyso-phosphatidylcholine (LPC) and lyso-phosphatidylethanolamine (LPE) species showed higher intensities in LXT followed by K326 and LXL, but lower intensities in BDH and LY (**Supplementary Figure S3**), suggesting positive relationships between these LPC and LPE species in tobacco leaf and the sensory quality of heated tobacco products. For flavonoids and tannins, BDH and LY had similar profiles, where most flavonoids and tannins were low in abundances except for 2-hydroxynaringenin and apigenin-6-C-fucoside (**Supplementary Figure S4**). In contrast, LXT, K326 and LXL had profiles containing higher abundances of many flavonols such as quercetin, kaempferol, dihydrokaempferol and their glycosyl derivatives. The heatmap of phenolic acids and organic acids was similar to that of flavonoids, where the abundances of most phenolic acids were higher in LXT, LXL and K326, whereas lower in LY and BDH (**Supplementary Figure S5**). The phenolic acids most unique to LY included maleoyl-caffeoylquinic acid, β-ureidoisobutyric acid and 2-O-caffeoylmalic acid.

## 4 Discussion

### 4.1 Tobacco leaf metabolites influenced the sensory quality of heated tobacco products

Tobacco leaf chemical compositions have been reported to highly influence the sensory quality of tobacco products (Weeks et al., 1992; Chou and Que Hee, 1994). Previous studies have particularly reported that volatile compounds of cured tobacco leaf influenced the taste and aroma of tobacco smoke and correlated with sensory quality of combustible tobacco products (Weeks et al., 1989). Volatile compounds such as β-damascenones, megastigmatrienone, ionone, cembratrieneol were identified as important evaluation indexes that were used to predict the quality and quantity of leaf aroma in combustible tobacco (Slaghenaufi et al., 2016; Yan et al., 2016; Popova et al., 2019). To enhance the aroma and sensory quality of combustible tobacco, numerous studies have focused on the regulatory mechanisms involved in modulating the biosynthesis of these carotenoid or diterpenoid degradation products (Shi et al., 2015; Zhang et al., 2015; Sui et al., 2018). However, studies on the relationships between tobacco leaf metabolites and sensory quality of heated tobacco products are limited. Chen et al. (2021) and Zhao et al. (2020) have evaluated the links between leaf chemistry and sensory attributes of heated tobacco, in which the levels of certain chemical class were summed from concentrations of all individual compounds. Effects of individual metabolites on sensory quality of heated tobacco remain largely open for investigation. Moreover, whether the compounds associated with good sensory evaluation in combustible tobacco can be used to grade sensory perception of heated tobacco remains to be investigated. In the study reported herein, the objective was to take a deep insight into the metabolome of tobacco leaf and characterize discriminating metabolites with strong contributions to the prediction of sensory perception of heated tobacco by OPLS-DA. Using non-targeted metabolomics to profile leaf metabolites of the five tobacco varieties, we were able to annotate 309 volatile and 1168 non-volatile metabolites. Higher abundances of most non-volatile compounds were observed in the higher sensory rated varieties (LXT, K326 and LXL) (**Figure 2B**), suggesting that the overall sensory quality of heated tobacco imparted by the non-volatile composition was generally perceived as positive. The variations in chemical profiles revealed by PCA and HCA indicated that leaf volatile and non-volatile metabolome annotated could be grouped and clustered by sensory quality ratings (**Figures 1**, **2**). These results indicated that classifications of sensory quality ratings were primarily influenced by leaf metabolome. The OPLS-DA supervised approach allowed the modeling of the sensory rating levels of five tobacco varieties based on volatile and/or non-volatile metabolites (**Figure 3**). Models were assessed for goodness of fit and predictive ability by calculating R^2^Y and Q^2^Y, respectively (Senizza et al., 2023). The model fit and predictive ability statistics of OPLS-DA model constructed using volatiles were comparable to the results from the model constructed using non-volatiles, suggesting that both volatile and non-volatile compositions of tobacco leaf contributed to the sensory perception of heated tobacco. Following VIP analysis and p-value together with fold-change analysis, 13 out of 309 volatiles and 345 out of 1168 non-volatiles were characterized as the discriminating compounds detected as either significantly higher or lower in tobacco varieties with higher or lower sensory ratings (**Table 2** and **Supplementary Table S5**). There were considerably more non-volatile compounds than volatile compounds with strong prediction of sensory quality of heated tobacco, which may further implicate greater influences of non-volatile compositions on the perception of sensory quality and higher efficacy of using non-volatile compounds for prediction of sensory quality at low heating temperatures.

### 4.2 Leaf metabolites able to discriminate tobacco varieties with higher and lower sensory ratings

Many volatile and non-volatile discriminating compounds that were identified by VIP analysis, p-value and fold-change individual values, are also substances associated with good sensory perception in combustible tobacco. For example, volatile compound β-damascenone (**Figure 4A**), non-volatile compounds glutamine derivative (L-glutamine-O-glycoside) (**Supplementary Figure S1**), scopoletins (**Supplementary Figure S2**), chlorogenic acids and neochlorogenic acids (**Supplementary Figure S5**) showed higher abundances in higher sensory rated varieties (K326, LXL and LXT) in conjunction with the VIP scores >1 and fold-change > 2 (**Table 2** and **Supplementary Table S5**). Beta-damascenones are a class of tobacco carbonyls produced from carotenoid degradation, and they were previously identified as the key volatile compounds positively correlated with tobacco aroma in combustible tobacco products (Popova et al., 2019). The pyrolytic products of aliphatic α-amino acid glutamine were reported to be present in the tobacco smoke, affecting the perceived sensory quality of combustible tobacco (Sharma et al., 2006). Scopoletin is commonly used as a chemical indicator of the aroma property of cured tobacco leaf (Yu et al., 2010) and it has also been found to have a significant positive correlation with aroma of heated tobacco product (Chen et al., 2021). Chlorogenic acids and its isomer neochlorogenic acids account for more than 50% of total phenolic acids in flue-cured tobacco (Sheen et al., 1969; Sheen, 1973), and act as precursors of aroma impact compounds in flue-cured tobacco leaf, providing typical tobacco aroma and rich taste in combustible tobacco products (Xie et al., 2018). They were also found to be positively associated with the aroma and overall sensory quality of heated tobacco in Chen et al. (2021). These compounds with positive relevance to the sensory evaluation in combustible tobacco also strongly contributed to the prediction of sensory quality of low heat tobacco. Therefore, if the previous work with combustible tobacco has a metabolomics profile, the variations of these compounds can be derived to predict the sensory quality of the same tobacco variety in heated tobacco, and vice versa. Flavonoids such as kaempferol, quercetin, kaempferol-3-glucoside and quercetin-3-rutinoside have previously been related to sensory quality in combustible tobacco, where they were reported to impart special flavor during tobacco curing and aging (Wu et al., 2022). Chen et al. (2021) quantified kaempferol and quercetin-3-rutinoside in different tobacco types and found positive correlation with physiological strength and taste of heated tobacco products. Consistent with previous sensory evaluation research, current study identified many flavonols and their glycosyl derivatives as strong positive predictors of sensory quality of heated tobacco (**Supplementary Figure S4**). As flavonoids and carotenoids are related to stress resistance, plant growth and development, crop quality, and so on, flavonoid and carotenoid biosynthesis in tobacco have been studied extensively (Shi et al., 2014; Shi et al., 2015a; Shi et al., 2015b; Wang et al., 2020b; Zhao et al., 2021). Therefore, components such as β-damascenone, chlorogenic acids, quercetin, quercetin-3-rutinoside can be breedable and tobacco lines varying on these constituents can be generated to further evaluate whether they would have higher sensory quality for combustible and low heat tobacco products. Moreover, these tobacco lines are proper genetic resources that might determine the efficacy of using only flavonoids and/or carotenoids to rank tobacco quality by sensory perception prior to actual processing and evaluation.

In addition to the compounds positively impacting the sensory quality of both combustible and heated tobacco, several volatile and non-volatile compounds that have a positive relevance with aroma and taste attributes in combustible tobacco did not strongly associate with the sensory ratings of heated tobacco. Volatiles including dihydroactinidiolide, 5-methyl furfural, 6-methyl-5-hepten-2-one and carotenoid-derived aromatic compounds such as megastigmatrienone and ionone were recognized as aroma-related components commonly used to predict the quantity of leaf aroma in combustible tobacco (Clark and Bunch, 1997; Slaghenaufi et al., 2016). However, they were not identified as discriminating compounds by the VIP scores and fold change analysis in the present study (**Supplementary Table S3**). Similarly, non-volatile compounds such as solanone and proline were not able to discriminate higher and lower rated tobacco varieties (**Supplementary Table S4**). Thus, these aroma-related volatile and non-volatile compounds in combustible tobacco have relatively week contribution to the prediction of sensory properties of heated tobacco. Interestingly, comprehensive metabolomics profiling followed by discriminant analysis allowed the discovery of some new relationships between leaf metabolites and sensory quality ratings of heated tobacco. Several lipid species were found to have positive influences on the perception of sensory quality of heated tobacco (**Supplementary Figure S3**), and therefore they should be considered together with other non-volatile metabolites when evaluating the applicability of tobacco varieties to heated tobacco products. LPC is a lysophospholipid consisting of one long hydrophobic fatty acid chain and one hydrophilic choline head group, attached to the glycerol backbone, and was found to enhance tobacco resistance to pathogen infection (Wi et al., 2014). LPE produced through hydrolysis of phosphatidylethanolamine by phospholipase A2 functions in the early stage of plant senescence and primes the plant immune system (Volz et al., 2021). Although the biological roles of these lipids have been investigated in tobacco, this is the first report of their positive contributions to the sensory perception of heated tobacco. These lipid species in tobacco leaf can be further investigated for their dynamic changes in physical and chemical properties at the temperatures of 200-350°C, and their interactions with the added agents such as glycerol to form the heated tobacco aerosol (Gasparyan et al., 2018) that might help understand their specific contributions to the sensory properties of heated tobacco. Moreover, many di-, tri- and oligosaccharides varied with the sensory rating levels (**Supplementary Figure S2**) and these reducing and non-reducing sugar molecules might be derived from the degradation of macromolecules such as starch and pectin during leaf curing (Tao et al., 2022). The carbonyls of reducing sugars can interact with amino acids to produce melanoid polymers (flavor compounds) through Maillard reactions, improving tobacco sweetness characteristics and aroma quality (Quan et al., 2020). Lower temperatures and humidity during air-curing process have previously been found to reduce sugar concentrations in air-cured tobacco varieties (Zhao et al., 2022). The lower abundances of these sugar molecules in the cured leaves of LY and BDH might therefore be attributed to the air-curing process.

## 5 Conclusions

Non-targeted metabolomics profiling and OPLS-DA together with fold-change values outlined volatile and non-volatile compounds that were correlated with the higher or lower sensory rating classes. Some compounds that were previously found in combustible tobacco associated with good sensory evaluation are the same as that with the low heat tobacco. Some new relationships between leaf metabolites and sensory quality of heated tobacco were found: several lipid species, and reducing and non-reducing sugar molecules were positively related to sensory quality ratings of heated tobacco. These discriminating volatile and non-volatile compounds can represent the first step for gaining insights into the chemical predictors for selecting tobacco varieties with desired sensory quality in heated tobacco.

## Data availability statement

The original contributions presented in the study are included in the article/**Supplementary Material**. Further inquiries can be directed to the corresponding authors.

## Author contributions

LZ and SS conceived the project and designed the study. LZ wrote the manuscript. SS, YT, YG performed the data analysis. ZS, LP and ZL contributed to data acquisition. BW supervised the research and reviewed the manuscript. All authors contributed to the article and approved the submitted version.

## Supplementary material

Supplementary Table 1 Volatile metabolites detected by GC-MS.

Supplementary Table 2 Non-volatile metabolites detected by UPLC-MS/MS.

Supplementary Table 3 Discriminant volatile metabolites identified by VIP (VIP>1.0) and p-value (p-value< 0.01).

Supplementary Table 4 Discriminant non-volatile metabolites identified by VIP (VIP>1.0) and p-value (p-value< 0.01).

Supplementary Table 5 Discriminant non-volatile compounds identified by VIP analysis and p-value together with fold-change cut off > 2 or < 0.5. The compounds highlighted in red were the top 50 discriminant non-volatiles ranked by t-test and used to construct the heatmap in **Figure 4B**.
