# Supplementary material for: Integrative analysis of sensory evaluation and non-targeted metabolomics to unravel tobacco leaf metabolites associated with sensory quality of heated tobacco
Source: Front Plant Sci. 2023 Feb 8;14:1123100. doi: 10.3389/fpls.2023.1123100 (PMC9944805; doi:10.3389/fpls.2023.1123100)
Supplement: Supplementary file 2 [file DataSheet_1.docx]

Supplementary Material

# Supplementary Figures


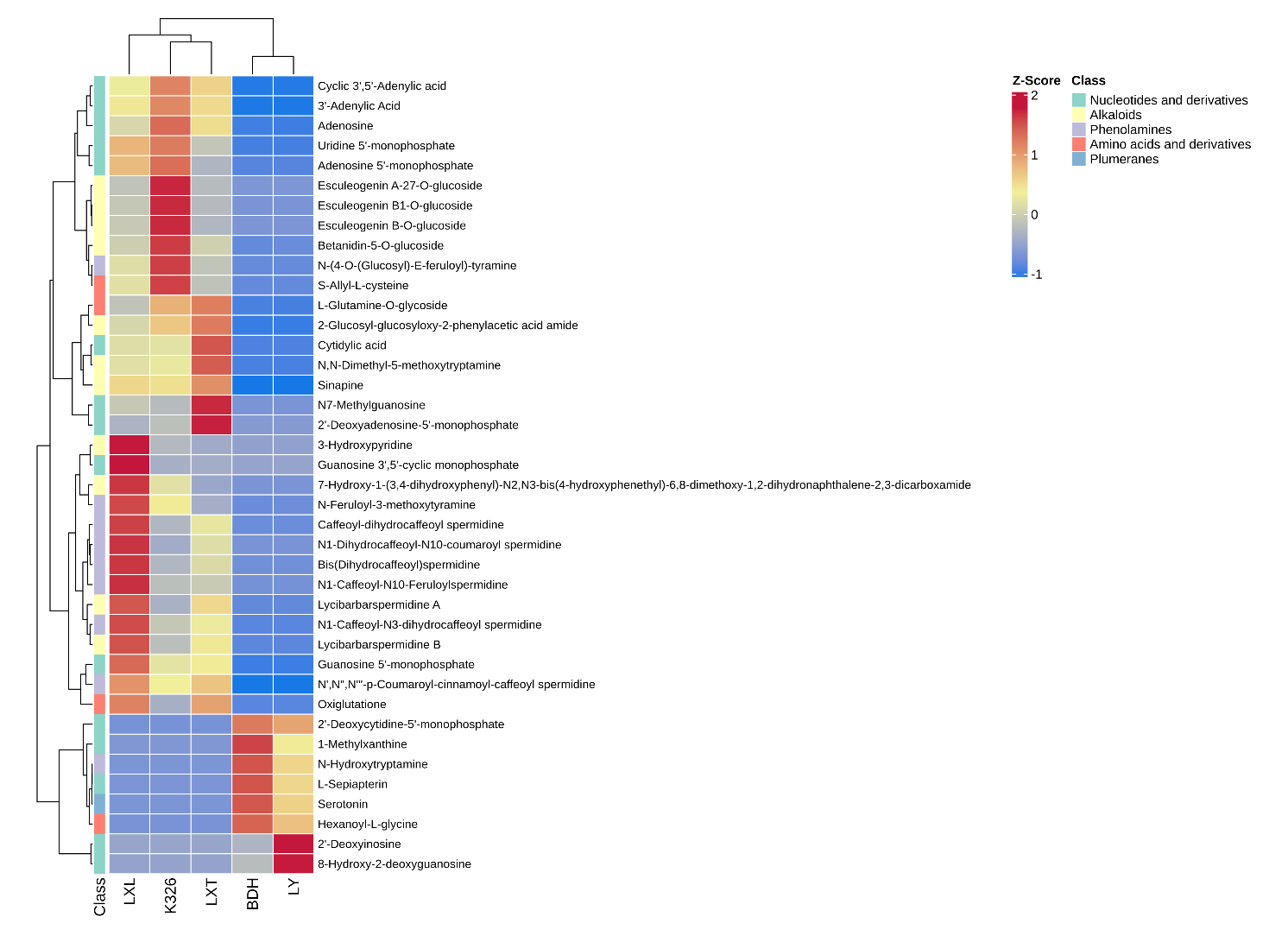
**Supplementary Figure S1.** Heatmap of 40 non-volatile compounds including 21 alkaloids, 15 nucleotides and derivatives, and four amino acids.

**
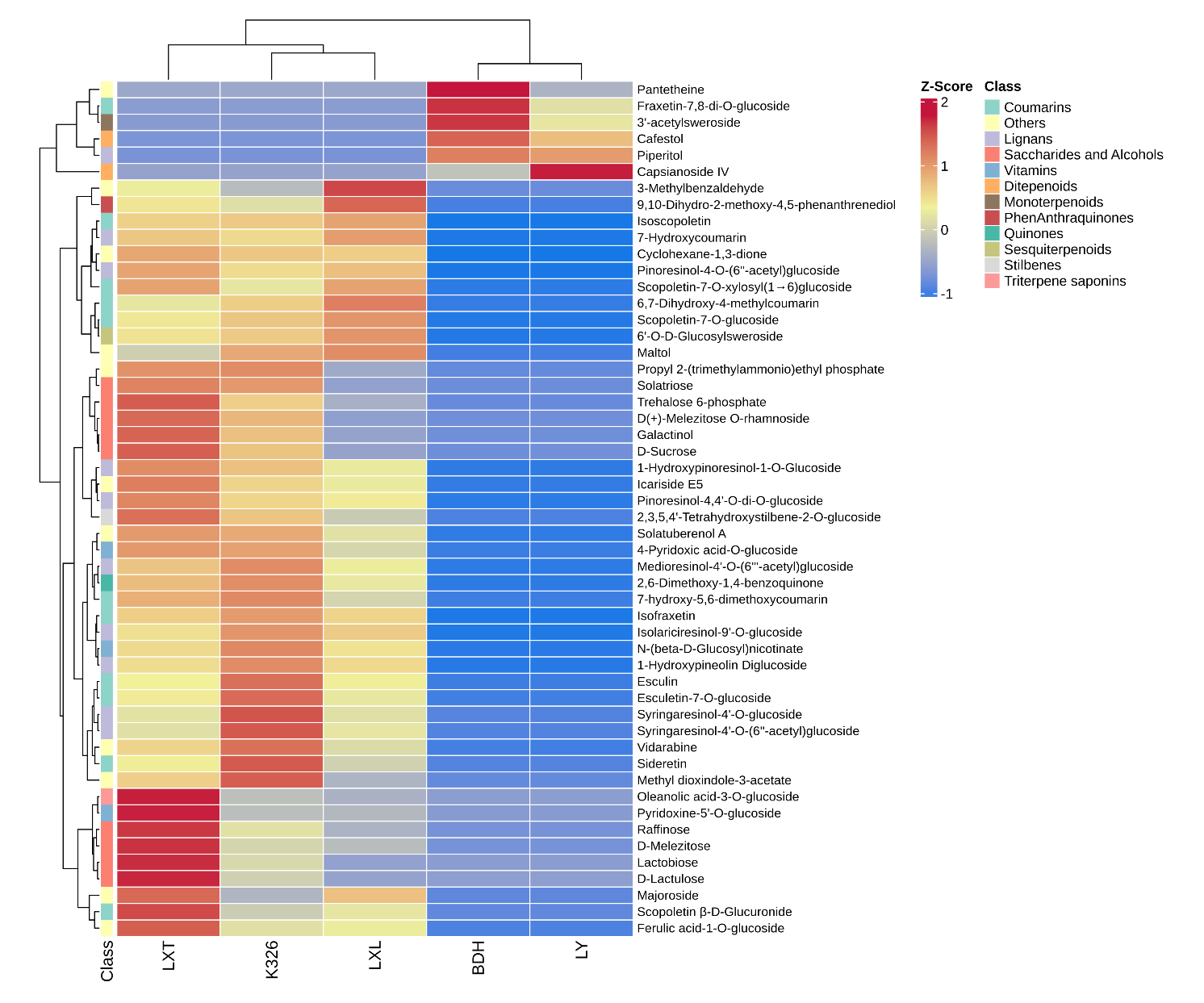
**

**Supplementary Figure S2.** Heatmap of 21 lignans, two quinones, five terpenoids, nine saccharides and alcohols, one stilbene, three vitamins and 11 other compounds.


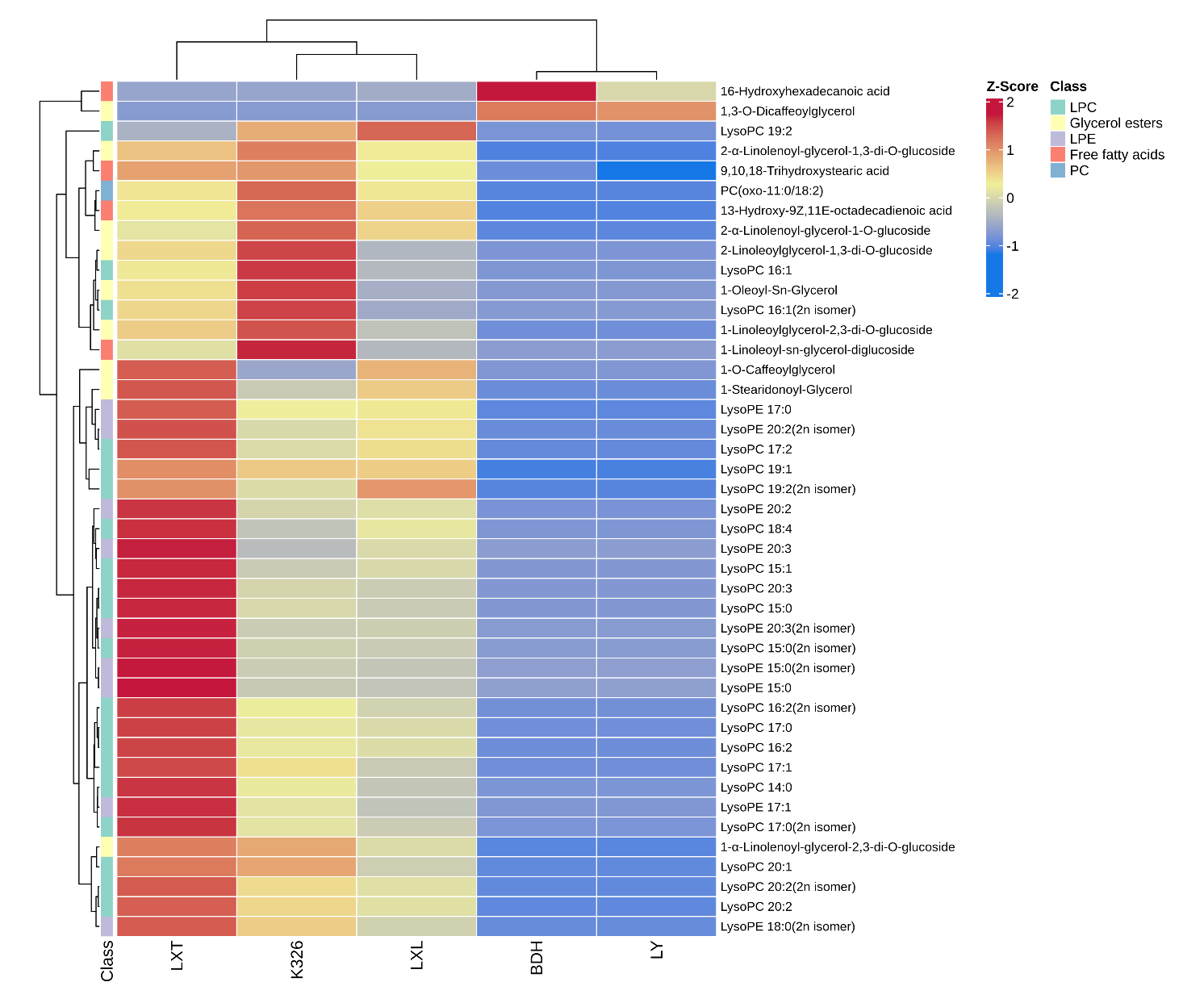


**Supplementary Figure S3.** Heatmap of 43 lipids.


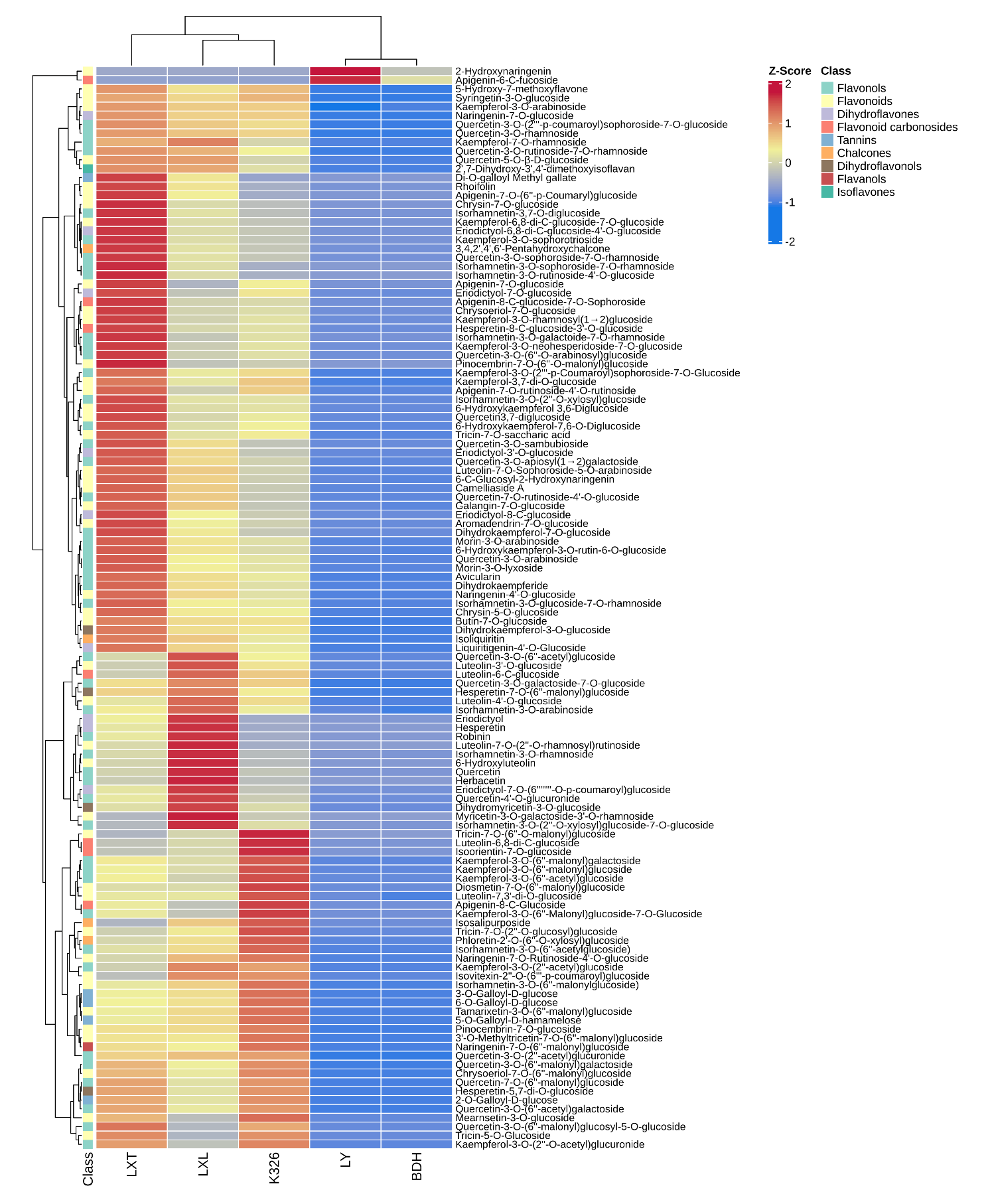


**Supplementary Figure S4.** Heatmap of 117 flavonoids and five tannins.


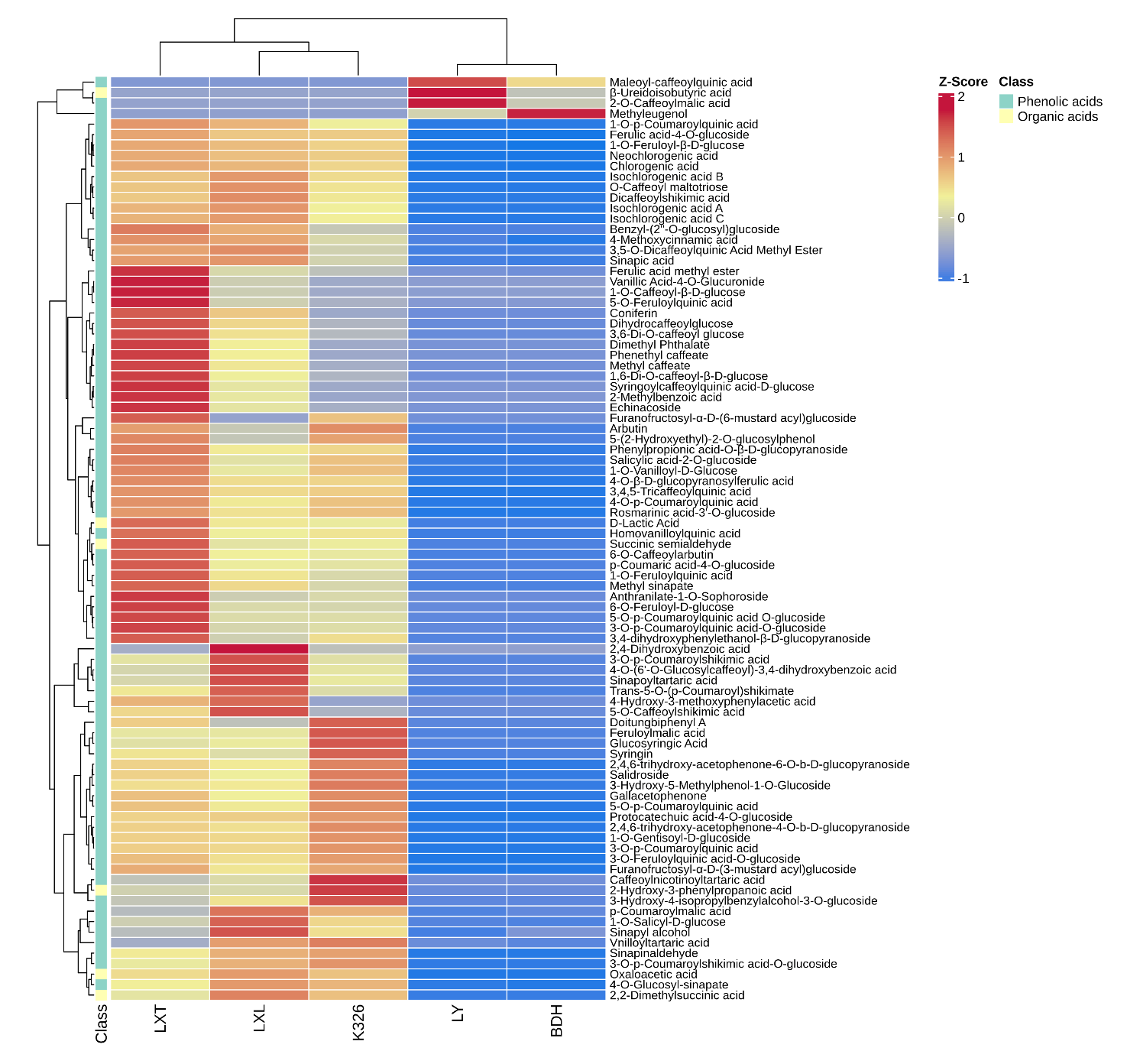


**Supplementary Figure S5.** Heatmap of six organic acids and 82 phenolic acids.
